# Titration of biologically active amyloid–β seeds in a transgenic mouse model of Alzheimer's disease

**DOI:** 10.1038/srep09349

**Published:** 2015-03-23

**Authors:** Rodrigo Morales, Javiera Bravo-Alegria, Claudia Duran-Aniotz, Claudio Soto

**Affiliations:** 1 Mitchell Center for Alzheimer's Disease and Related Brain Disorders, Department of Neurology, The University of Texas Houston Medical School, Houston, TX 77030; 2 Universidad de los Andes, Facultad de Medicina, Av. San Carlos de Apoquindo 2200, Las Condes, Santiago, Chile

## Abstract

Experimental evidence in animal models suggests that misfolded Amyloid-β (Aβ) spreads in disease following a prion-like mechanism. Several properties characteristics of infectious prions have been shown for the induction of Aβ aggregates. However, a detailed titration of Aβ misfolding transmissibility and estimation of the minimum concentration of biologically active Aβ seeds able to accelerate pathological changes has not yet been performed. In this study, brain extracts from old tg2576 animals were serially diluted and intra-cerebrally injected into young subjects from the same transgenic line. Animals were sacrificed several months after treatment and brain slices were analyzed for amyloid pathology. We observed that administration of misfolded Aβ was able to significantly accelerate amyloid deposition in young mice, even when the original sample was diluted a million times. The titration curve obtained in this experiment was compared to the natural Aβ load spontaneously accumulated by these mice overtime. Our findings suggest that administration of the largest dose of Aβ seeds led to an acceleration of pathology equivalent to over a year. These results show that active Aβ seeds present in the brain can seed amyloidosis in a titratable manner, similarly as observed for infectious prions.

Brain deposition of amyloid-β (Aβ) aggregates is a hallmark feature of Alzheimer's disease (AD). Accumulation of misfolded Aβ has been linked to cell and synaptic toxicity in both *in vitro* and *in vivo* models^1^,^2^,^3^. The most promising therapeutic strategies aimed to combat AD principally focus to modify the production and aggregation of this peptide^4^,^5^.

Protein aggregates sharing similar biophysical properties to those formed by Aβ in AD are found in various other diseases, termed Protein Misfolding Disorders (PMDs). PMDs include several neurodegenerative disorders, such as Parkinson's and Huntington's diseases, and prion diseases or transmissible spongiform encephalopaties (TSEs), among others^6^. TSEs are so far the only PMDs considered to be infectious. The infectious agent in TSEs, known as prion, is thought to be composed exclusively by the misfolded form (PrP^Sc^) of a normally synthesized protein, termed PrP^C^ (Ref. 7). The mechanism of prion transmission is best accounted by the seeding/nucleation model^8^,^9^,^10^, in which aggregates or “seeds” of the infectious protein are able to recruit monomeric PrP^C^ into the growing aggregate, where they acquire the misfolded conformation and can serve to further replicate prions. As extensively reported, the formation of amyloid aggregates in PMDs also follows the seeding/nucleation model, and therefore these aggregates have the potential for prion-like transmission^8^,^10^.

Several studies have shown that Aβ and other disease-associated misfolded protein aggregates are experimentally transmissible *in vivo*^11^,^12^,^13^,^14^,^15^,^16^,^17^,^18^,^19^. However, the lack of epidemiological data showing inter-individual transmission in other PMDs besides TSEs, suggests that prion-like mechanisms mostly operate to spread these deleterious aggregates between different tissues and cells within the affected individual. In the specific case of Aβ, most of the experiments have been performed by accelerating spontaneous deposition of aggregates produced by overexpression of mutant genes^11^,^12^,^13^,^14^,^16^,^20^. Nevertheless, some experiments have shown induced aggregation in animal models that normally do not develop amyloid deposits during their natural lifespan^15^,^21^, a fact approaching better to the situation in naïve prion infected animals.

One of the important characteristics of the prion agent is that its capacity to transmit pathology is directly proportional to the quantity of the misfolded protein inoculated and that transmission can be observed even after administration of highly diluted concentrations of prion-infected brain extracts. Titration of infectious prions is useful to estimate the load of infectious agent in specific samples^22^, as well as to quantitatively determine the therapeutic effect of candidate drugs or treatments^23^. The main goal of this study was to titrate biologically active Aβ seeds *in vivo* in a similar way as performed for infectious prions.

## Results

**Figure 1:**
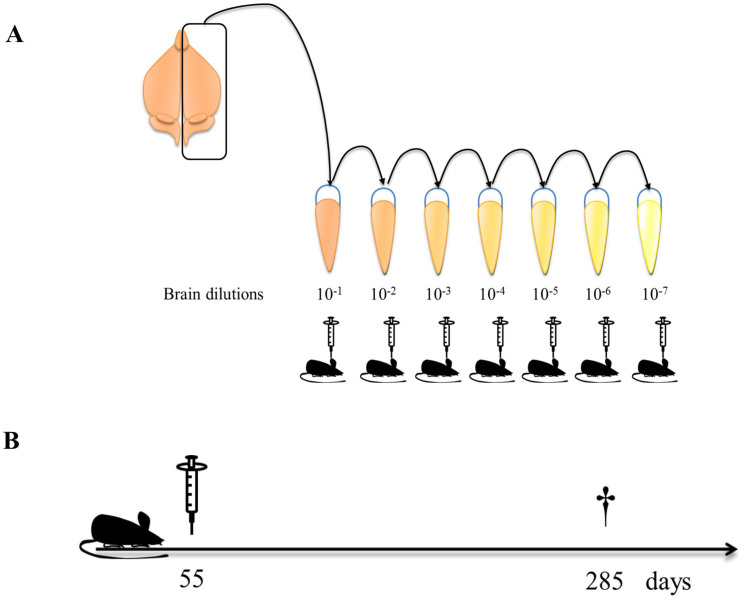
Experimental Strategy for injection of A**β** seeds at different concentrations in tg2576 mice. (A) Brains from four 18–20 months old tg2576 mice harboring extensive Aβ accumulation were homogenized at 10% w/v (10^−1^ dilution), pooled and serially diluted 10-folds in PBS. Samples were injected intra-cerebrally into tg2576 mice as described in Methods. (B) Timeline describing mice's age at injection and sacrifice. This figure was drawn by J.B.-A.

**Figure 2:**
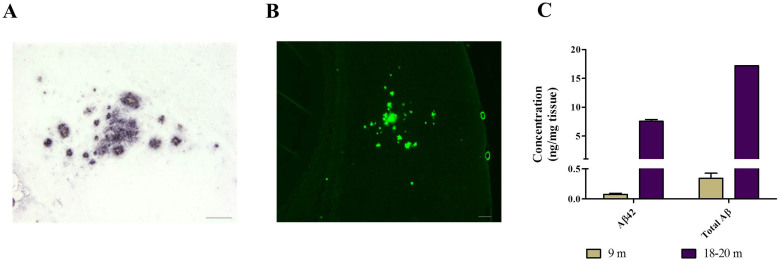
Characterization of the inoculum. 10% w/v homogenates were prepared from four 18–20 months old tg2576 animals and materials were pooled into a single stock. These brains carried large amounts of Aβ aggregates (A) which were also reactive to ThS (B). Pictures in (A) and (B) depict hippocampal and cortical regions of two different animals, respectively. Bars in both cases represent 100 μm. (C) The pooled tissue homogenate was subjected to a serial fractionation procedure (see Methods section) and the amount of aqueous insoluble Aβ_42_ or the levels of the total forms of insoluble Aβ were measured and compared to brains from young transgenic mice (9 months old) displaying little aggregation. Measurements were done in duplicate and data shown as average ± standard error of the mean.

Several previous experiments have shown that brain amyloidosis can be accelerated after intra-cerebral (i.c.) injection of misfolded Aβ aggregates in transgenic mouse models of AD^11^,^12^,^13^,^14^,^15^,^16^,^20^. However, a careful characterization of dose-dependent seeding efficiency and the minimum amount of seeds able to generate a significant change compared to non-treated animals has not been explored. For that purpose, we i.c. injected ~55 days old tg2576 mice with different dilutions of a pool of brain extracts obtained from 18–20 months old animals from the same transgenic line (Figure 1A). Animals were sacrificed at 285 days old (when scattered/small deposits are observed in untreated mice) and brains collected for immunohistochemical analyses to assess Aβ deposition (Figure 1B). Independent dilutions of this material showed a linear degree of decrease in terms of Aβ concentration (Supplementary Figure 1). First, we characterized the inoculum used for this study using conventional histological and biochemical techniques. As observed in Figure 2, contralateral brain hemispheres (respect to the ones used to prepare the inoculum) contained large amounts of Aβ aggregates that were reactive to Thioflavin S (ThS). Additionally, we observed that the inoculum contained substantially larger concentrations of PBS-insoluble Aβ aggregates when compared to younger (9 months old) animals (Figure 2C).

**Figure 3:**
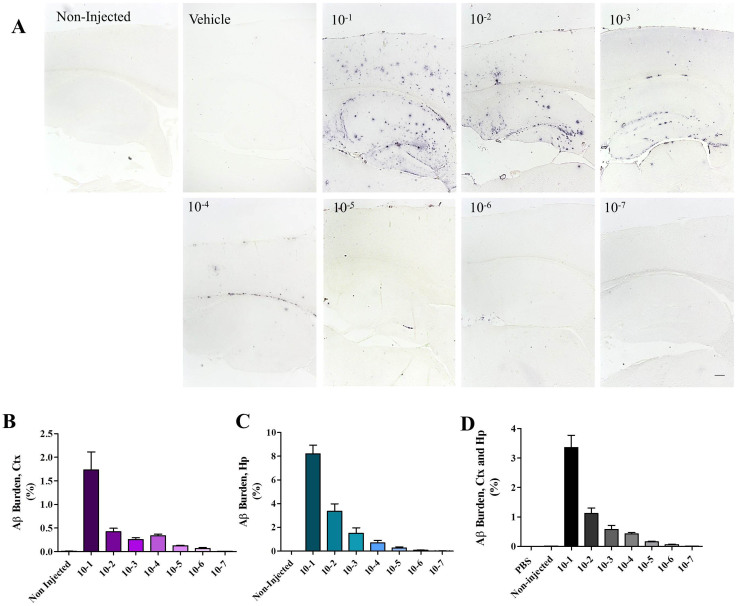
Dose-dependent acceleration of cerebral amyloid pathology by exogenous administration of A**β** seeds. Tg2576 mice were injected with different dilutions (10^−1^ to 10^−7^) of a brain homogenate containing large amounts of Aβ seeds. Animals were sacrificed at 285 days old and amyloid burden in cortex (Ctx) and hippocampus (Hp) was measured as described in Methods. (A) Representative pictures of cortex and hippocampus of non-treated- and treated-mice injected with different dilutions of the inoculum or the vehicle (PBS). Numbers at the top-left of each picture represent dilution regarding the brain. Bar at the low-right side of the “10^−7^” picture denotes 200 μm and is representative for all pictures in this panel. Aβ burden in cortex (B), hippocampus (C), or both areas combined (D) was expressed as the area stained by the 4G8 antibody versus the whole area analyzed, and expressed as a percentage. The statistical analysis of this data, including the exact P value is included in Table 1.

Inoculum, prepared as a 10% w/v homogenate, was injected directly or at different 10-fold dilutions ranging from 10^−2^ to 10^−7^ (Figure 1A). Animals were sacrificed at 285 days old and brain slices stained using anti-Aβ antibodies. The burden of signal in cortex and hippocampus was measured in each case and compared to the one obtained in non-challenged mice sacrificed at the same age. When compared to non-treated mice, we observed a dose-dependent increase in the Aβ burden in almost all injected groups. The sole exception was found for animals treated with the 10^−7^ brain dilution (Figure 3 and Table 1). Importantly, animals injected with the same volume of vehicle (PBS) showed a very small and similar degree of amyloid pathology when compared to non-treated animals (Figure 3A). This is similar to our previous results obtained after injection with brain extracts from non-AD subjects or PBS^20^, as well as samples where Aβ or misfolded proteins have been specifically removed^12^,^24^.

Amyloid deposits were mostly located in hippocampus and cortex (Figure 3), although scarce aggregation was also observed in the thalamus and olfactory bulb of animals injected with the most concentrated inoculum (Supplementary Figure 2). As displayed in Table 1, the higher concentration of Aβ injected in these mice increased the burden of Aβ aggregates over 480 times compared to non-injected animals. Interestingly, the increase of amyloid deposition due to exogenous seeding was observed even after diluting the brain 1,000,000-folds, resulting in a burden ~9 times higher than in non-injected subjects (Table 1). The extent of Aβ deposition measured histologically in both cortex and hippocampus decreased proportionally with the dilution of the material injected (Figures 3B–D).

**Figure 4:**
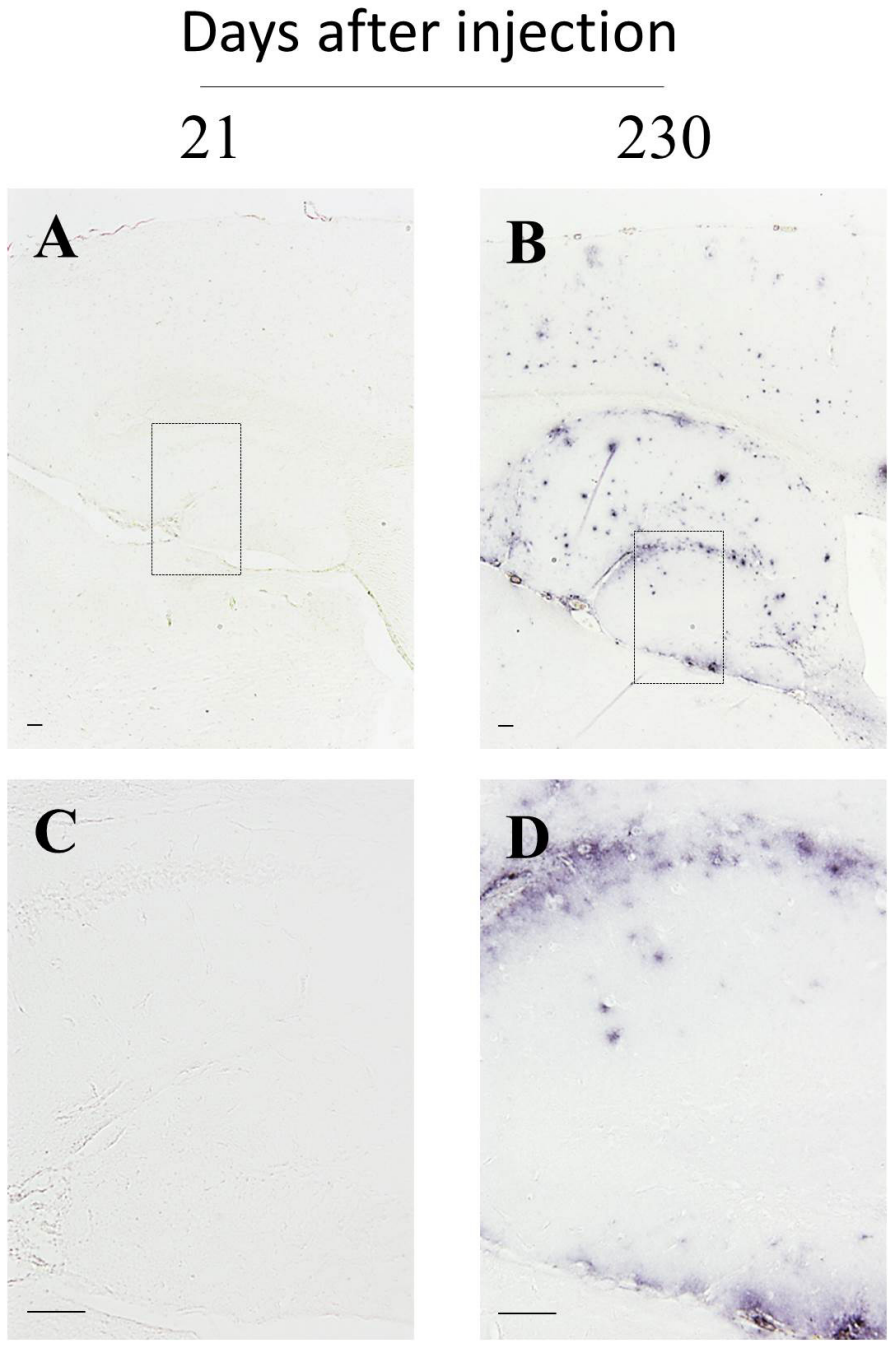
Absence of Aβ accumulation in animals injected with high concentration of Aβ seeds and sacrificed at short time points. Tg2576 animals injected with a highly concentrated inoculum (10^−1^) were sacrificed at 21- (A, C; n = 5) or 230-days (B, D; n = 6) after injection (76- or 285-days old, respectively). Pictures are representative of animals in both groups. (C) and (D) are amplifications of the areas depicted in (A) and (B), respectively. Lines at the bottom-left of pictures are representative of 100 μm (panels A and B) and 50 μm (panels C and D).

To confirm that the staining observed corresponded to accumulation of endogenous Aβ aggregates seeded by administration of exogenous material and not to the seeds themselves, we injected tg2576 mice with the most concentrated (10^−1^) inoculum and sacrificed the animals 21 days later. Brains from these animals were compared to the ones from mice receiving an equivalent inoculum but sacrificed 230 days later (285 days old) as described for the main experiment. We did not observe presence of anti-Aβ staining in animals sacrificed at the shorter time point (Figure 4), even when several brain slices at different distances from the injection site were analyzed.

**Figure 5:**
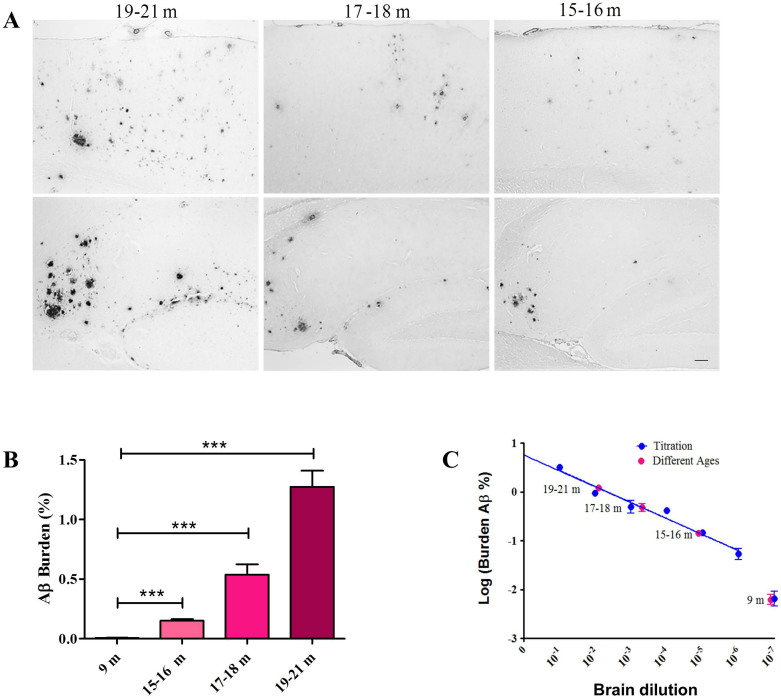
Spontaneous Aβ pathology in aged tg2576 mice. (A) Representative pictures of cerebral cortices (upper panels) and hippocampi (lower panels) of non-treated tg2576 mice sacrificed at different time points. Black line at the lower right panel represents 100 μm and is representative for all pictures in this figure. (B) Cortical and hippocampal Aβ burden in animals sacrificed at the designated time points. (C) The logarithm of Aβ burden in animals injected with different dilutions of brain homogenate (blue dots, data obtained from Table 1) was plotted versus the dilution of the inoculum administered. A linear correlation was observed (blue line, r^2^ = 0.9672). The data of the logarithm of Aβ burden in non-treated animals sacrificed at different time points (pink dots) was added to the graph to facilitate the comparison with the “Aβ titration curve”. For this purpose the dots were put in the line at the experimentally obtained Aβ burden (y-axis), except for the case of 9 months old animals in which the Aβ burden was outside the regression curve. Data in (C) is expressed as averages ± SEM. Data obtained in animals injected with the 10^−7^ inoculum dilution was not considered in the linear regression. 10, 9 or 10 mice were used in the 15–16, 17–18 or 19–21 months groups, respectively.

In order to extrapolate our results to the biological setting, and taking advantage that the spontaneous accumulation of amyloid deposits in these transgenic mice progressively increases with time, we sacrificed non-treated animals at different time points and measured the amyloid burden in their brains. Mice were sacrificed either at 15–16, 17–18 or 19–21 months old and brain slices analyzed histologically for the presence of Aβ aggregates (Figure 5A). The amyloid burden was calculated and plotted against the animal age (Figure 5B). As expected, aged mice showed extensive deposition of Aβ principally in the cortex and hippocampus and amyloid lesions increased overtime (Figure 5B). Interestingly, when this data was overlapped into the “titration curve” generated with the results of the transmission experiment with different dilutions of brain extracts, a positive correlation was observed between the increase of amyloid burden produced by aging and by transmission (Figure 5C). As an example, untreated mice from the 19–21 months old group showed an Aβ burden similar to the one observed in mice induced with the 10^−2^ dilution of the brain. As expected, younger untreated animals showed Aβ burden equivalencies consistent with induction using higher dilutions of the inoculum. Interestingly, none of the untreated mice, even at advanced age (21.3 months), generated an extent of Aβ deposition as high as the one induced by the 10% brain extract. At the dilutions tested, we were not able to observe a saturation of the amyloid inducing activity.

**Figure 6:**
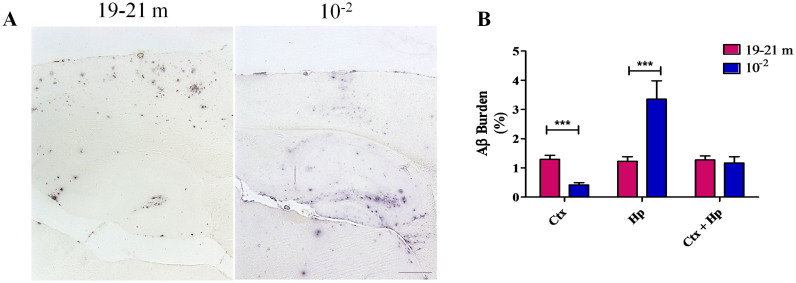
Differential brain distribution of Aβ deposits in amyloid treated and aged tg2576 mice. (A) Representative 4G8 stained brain slices from a non-treated animal sacrificed at 19–21 months old (left) and a mice injected with Aβ aggregates (10^−2^ inoculum dilution) and sacrificed at ~9,5 months old (285 days). Black horizontal line at the bottom of the right picture represents 150 μm and applies to both pictures. (B) Aβ burden in cortex (Ctx), hippocampus (Hp) and both areas together in mice from the groups mentioned in (A). Results are represented as means ± SEM. ***p < 0.01.

Although similar total Aβ burdens were observed in some induced vs. aged groups of transgenic animals, the brain regional distribution of Aβ aggregates found in each case was different. In our experiments, induction of Aβ aggregation was performed by i.c. administration of seeds into the hippocampus. Interestingly, a higher burden of Aβ deposits was found in this anatomical structure compared to old animals having the same burden considering cortex and hippocampus together (Figure 6). On the contrary, aged animals generated a higher cortical burden of deposits when compared to induced subjects. This data suggest that the distribution of aggregates depends not only on the nature of the transgenic animal, but also on where the seeds are originally placed.

It is thought that in the brain of AD patients accumulation of Aβ deposits leads to changes in the phosphorylation and aggregation of the Tau protein, resulting in the formation of neurofibrillary tangles. To investigate whether extensive deposition of Aβ aggregates induced tau hyperphosphorylation, we stained brain slides from animals in various conditions with an antibody that recognizes hyperphosphorylated tau. Under our experimental conditions, we were unable to detect hyperphosphorylated tau in the brain of animals injected with the most concentrated brain extract or even in non-injected old animals (Supplementary Figure 3).

## Discussion

During recent years there has been great interest to explore the putative prion-like features of protein aggregates involved in PMDs other than TSEs. Among them, tau and Aβ have taken a highlight place due to their involvement in AD, the most common type of senile dementia. Previous *in vitro* and *in vivo* experiments exploring the prion-like features of Aβ aggregates, added to the current knowledge gathered on TSE-prions, have posited a novel mechanism for the spreading of these deleterious molecules during the disease. A strong debate currently exists on whether Aβ and other misfolded protein aggregates are prions in the sense of their mechanism of transmission, principally due to the lack of evidence of natural inter-individual transmission. So far, no epidemiological data suggest that misfolded Aβ is able to transmit disease between individuals. However, it is important to highlight that human prion diseases are not easily transmissible by standard routes and the onset of clinical disease normally occurs after years or decades of a silent incubation period.

Several properties are often observed associated to prions and are considered necessary for them to behave as infectious agents^8^,^10^,^25^. Some of them have been successfully reported for Aβ aggregates, such as the reproducible induction of pathology in appropriate hosts^11^,^12^,^15^, transmission by peripheral routes^13^, removal of induction activity by depleting the misfolded form of the protein^12^,^24^, existence of conformational strains of the agent^26^,^27^,^28^, transmission of disease-associated protein misfolding by samples from pre- or a-symptomatic individuals^20^, serial propagation in animal bioassays^27^, and acceleration of pathology by preparations composed of purified/synthetic misfolded forms of the protein^16^. Nevertheless, other typical PrP^Sc^ properties have still not been explored, feeding the current controversy on whether misfolded Aβ is actually a prion. Among the non-explored prion features of misfolded Aβ we can include the titration of the agent, which is a common test to measure the specific PrP^Sc^ transmissible activity. Prions can efficiently maintain infectivity even after subjecting infected samples (i.e. brain tissues) to high dilutions. The purpose of the current study was to titrate biologically active Aβ seeds *in vivo* and define the minimum dose capable to induce pathological changes. The experiments described in this article were done with tg2576 mice, which is a transgenic line that over-express the human Amyloid Precursor Protein (APP) harboring the Swedish mutation and as a consequence starts developing the first visible Aβ aggregates at ~9 months old^29^. Unfortunately, extensive deposition of Aβ does not cause obvious clinical signs, nor death as observed for prion infected subjects. Due to these limitations, we sacrificed animals at 285 days old (230 days after injection) when Aβ deposition is barely detectable in untreated animals. The disease onset and progression was measured by the burden of Aβ deposits in treated animals compared to non-treated controls sacrificed at the same age.

Exogenous administration of high concentrations of Aβ seeds dramatically accelerated pathological changes, reaching a stage which is more severe than in even very old non-treated transgenic subjects (Figures 3 and 5). By analyzing brain slices of animals sacrificed 21 days after treatment we confirmed that amyloid pathology observed in our experimental endpoint (230 days after injection) corresponded to *de novo* generation of Aβ aggregates and not to the original inoculum (Figure 4). A significant acceleration of brain amyloidosis was induced even when diluting the brain material one million times (Table 1). Calculation of the initial load of Aβ aggregates in the stock inoculum (Figure 2C), enable to estimate that a 10^−6^ dilution contain ~343 fg of Aβ aggregates, which would correspond to ~45 × 10^6^ molecules of Aβ monomer. Interestingly, this number approaches the estimation of the last infectious unit for 263K hamster prions, a widely used experimental agent in prion research. Indeed, the minimum amount of 263K prions able to cause infectivity is estimated at ~50 fg (corresponding to a 10^−8^ brain dilution)^30^. However, 263 K is one of the fastest and most efficient prion strains, and indeed the last infectious brain dilutions for other prion strains are in the range of 10^−5^ to 10^−7^ (Ref. 31). It is important to mention that although initial dilutions of the inoculum showed a linear decrease of Aβ (Supplementary figure 1), we were unable to follow whether this linear decrease of seeds continued to the higher dilutions injected in this experiment. Due to the heterogeneous nature of amyloid aggregates, we acknowledge that slightly different results could be obtained when independently made dilutions are injected in mice.

The availability of a “titration curve” for the transmissibility of Aβ aggregates may be useful to assess the specific burden of Aβ seeds present in different tissues or biological fluids, as well as to quantify the effect of disease modifying strategies directed to understand or combat AD. Interestingly, the degree of the induction of amyloid pathology obtained with distinct dilutions of the brain extract was comparable to that observed in non-treated animals sacrificed at different ages (Figure 5C). In our study we found that i.c. administration of a 1% brain extract (10^−2^ dilution) induced amyloid deposition to a comparable extent of that observed spontaneously in these transgenic mice at around 19–21 months of age. In other words, our results suggest that injection of ~3.43 ng of aggregated Aβ (the amount estimated in a 10^−2^ dilution) is able to accelerate brain amyloid pathology in an extent equivalent to 10–12 months. However, and in agreement with a previous report^32^,^33^, seeding in induced animals was shifted towards the injection site when compared to aged non-treated transgenic mice. Studies using purified Aβ preparations obtained from synthetic, recombinant or mammalian origins would be needed to investigate if pure Aβ aggregates have the same potency as brain extracts. These studies will allow to understand whether Aβ seeding activity depends only on Aβ structures or there are other molecules or co-factors that play a role in transmission, as has been suggested for infectious prions^34^. Future studies, in appropriate animal models, could also help to define the minimum amount of Aβ aggregates able to trigger the cascade of pathological events observed in the disease, including tau hyper-phosphorylation, formation of neurofibrillary tangles, inflammation, synaptic and neuronal toxicity.

In conclusion, in this study we show that biologically active Aβ seeds are titratable and that the minimum amount able to induce pathological changes is in the same range as described for infectious prions. In the light of this data, it is important to further explore the prion-like properties of Aβ aggregates in order to fully understand how these particles spread and finally exert their deleterious effects in the brain. Comparing the mechanisms of misfolding and aggregation of Aβ and prions may be useful to design novel diagnostic and therapeutic strategies directed to attack the spreading of pathological alterations in AD.

## Methods

### Animals

Tg2576 transgenic mice express the human amyloid precursor protein gene harboring the Swedish mutation^29^. These animals start to develop cerebral Aβ deposits at ~9 months old. Mice were housed in standard conditions (22°C, 12 h dark/light cycles, food and water *ad libitum*) as groups of no more than five mice per cage. All animal procedures described in this article were in agreement with the regulations of the Center of Laboratory Animal Medicine and Care (CLAMC) and Animal Welfare Committee (AWC) of the University of Texas Medical School at Houston. 4–10 animals (random mixtures of males and females) were used per experimental group as indicated in Table 1 or the respective figure legends.

### Preparation of inocula

Brain halves from four 18–20 months old tg2576 mice (containing extensive amyloid deposits, Figure 2) were homogenized at 10% w/v in Phosphate Buffer Saline (PBS, MP Biomedicals, Santa Ana, CA, USA) containing a cocktail of protease inhibitors (Roche Diagnostics GmbH, Mannheim, Germany). The materials were pooled and the resulting sample (denoted as 10^−1^) was serially diluted in 10-fold dilutions using the same buffer until reaching a 10^−7^ dilution regarding the brain. Resulting inocula were stored at −80°C until used for animal injection.

### Serial extraction and ELISA measurement of Aβ

200 μL of 10% w/v brain homogenates from the original inoculum (10^−1^) or 9 months old tg2576 mice were ultra-centrifuged at 100,000 × g for 1 h at 4°C using a L100K Beckman-Coulter ultracentrifuge (Beckman-Coulter, Brea, CA, USA). Supernatants were discarded and pellets resuspended in 200 μL of 70% formic acid. Samples were sonicated in a bath sonicator and centrifuged for 30 min in the same conditions listed above. Supernatants were collected, diluted 20 times in 1 M Tris buffer (pH 11) and snap frozen in liquid nitrogen. Resulting samples were stored at −80°C until tested for Aβ concentration using an Aβ_42_ specific ELISA kit (Invitrogen, Carlsbad, CA, USA) or an ELISA kit detecting total Aβ (IBL, Aramachi, Japan). Extractions and ELISA measurements were performed two times in duplicates by two different investigators. In order to assess the linearity of the diluted material, a 1.25% brain homogenate sample of the original inoculum was 2-fold serially diluted in PBS three independent times. Resulting samples were diluted 1/5 in 88% formic acid and further diluted 20 times in 1 M Tris buffer for neutralization. Aβ concentration was measured by ELISA as described.

### Animal procedures

~55 (50–55) days old tg2576 mice were i.c. injected with different tg2576 brain homogenate dilutions (Figure 1). Injections were performed stereotaxically in the hippocampus (both hemispheres) of anesthetized animals using the following coordinates from bregma: anteroposterior, −1.8 mm; medio-lateral, ±1.8 mm; dorso-ventral, −1.8 mm. 10 μL of sample were administered per injection site using a Hamilton syringe. Mice were sacrificed by CO_2_ inhalation at 21 or 230 days after injection and tissues collected for histological analyses. Additional groups of untreated tg2576 animals were sacrificed at different time points ranging from 9 to 21 months old.

### Histological studies

Formalin fixed brains were dehydrated for paraffin inclusion. Brains were serially sliced in 10 μm thick sagittal sections from the midline. 5 slices (one every tenth slice) per animal were processed for immunohistochemistry. Briefly, sections were deparaffinazed and the endogenous peroxidase activity was blocked with 3% H_2_O_2_/10% methanol in PBS, for 30 min. Then, brain sections were incubated in 85% formic acid for 5 min for antigen retrieval. Sections were incubated (overnight, room temperature) with the mouse 4G8 antibody (Covance, Princeton, NJ, USA), which recognizes the 17–24 sequence of Aβ, diluted 1:1000 in PBS/0.02% Triton-X100 (Sigma, St. Louis, MO, USA), or anti-hyperphosphorylated tau AT8 antibody, diluted 1:200 in the same buffer (Pierce). After washing with PBS, sections were incubated for 1 h with an HRP-linked secondary sheep anti-mouse antibody at a 1:500 dilution (GE Healthcare, Little Chalfont, UK). Peroxidase reaction was visualized using DAB Kit (Vector, Burlingame, CA, USA) following the manufacturer's instructions. Finally, sections were dehydrated in graded ethanol, cleared in xylene, and cover-slipped with DPX mounting medium (Innogenex, San Ramon, CA, USA). ThS staining was performed by incubating tissue slices with a ThS (Sigma, St. Louis, MO, USA) solution (0.1% in 50% ethanol) for 15 minutes after deparaffinization. After incubation, sections were washed for 2 minutes in 80% ethanol, dehydrated and cover-slipped as described.

### Image analyses of brain slices

For quantification, brain slices were examined under a DMI6000B microscope (Leica, Buffalo Grove, IL, USA) and image analysis was performed using the ImageJ software (National Institutes of Health, Bethesda, MD, USA). 4G8 burden was defined as the antibody labeled area in each tissue slice per total area analyzed (hippocampal and cortical areas only), and expressed as percentage.

### Statistical Analysis

Kolmogorov-Smirnov (K-S) statistic test was used to confirm normal distribution of the data. According to their distribution, Student's t-test (10^−1^ to 10^−5^) or Mann-Whitney U-test (10^−6^ and 10^−7^) were used to compare Aβ burden in injected and non-injected animals. The values are expressed as means ± SEM. Data was analyzed using the Graph Pad prism software, version 5.0. Statistical differences were considered significant for values of P < 0.05.

## Author Contributions

R.M. designed the experiments, performed most of the animal manipulations, analyzed the data, and wrote the manuscript. J.B.-A. performed most of the histological analyses and prepared the final version of the figures. C.D.-A. participated in some animal and histological analyses. C.S. designed the experiments, supervised the project and wrote the manuscript.

## Supplementary Material

Supplementary Information Supplementary figures

## Figures and Tables

**Table 1:** Amyloid pathology in injected versus non-treated animals

Inoculum dilutions	n^a^	4G8 burden (mean ± sd)	Fold increase	P value
10^−1^	6	3.357 ± 1.028	482.2582962	<0.0001^b^
10^−2^	6	1.118 ± 0.4971	160.6091079	0.0001^b^
10^−3^	5	0.5775 ± 0.3182	82.96221807	0.0008^b^
10^−4^	5	0.4274 ± 0.1039	61.39922425	<0.0001^b^
10^−5^	7	0.1553 ± 0.066	22.31001293	<0.0001^b^
10^−6^	6	0.0638 ± 0.0424	9.171096107	<0.0011^c^
10^−7^	4	0.0086 ± 0.0042	1.238902457	0.3048^c^
Non-injected	6	0.0069 ± 0.0035	---	---

^a^n = number of animals per experimental group.

^b^Student’s t-test.

^c^Mann Whitney U-test, because data did not follow a normal distribution, so t-test could not be applied.
