# Supplementary material for: Titration of biologically active amyloid–β seeds in a transgenic mouse model of Alzheimer's disease
Source: Sci Rep. 2015 Mar 23;5:9349. doi: 10.1038/srep09349 (PMC4399520; doi:10.1038/srep09349)

SUPPLEMENTARY INFORMATION

Titration of biologically active amyloid–β seeds in a transgenic mouse model of Alzheimer's disease

Rodrigo Morales[1,#], Javiera Bravo-Alegria[1,2,#], Claudia Duran-Aniotz[1,3] and Claudio Soto[1,*]

Supplementary Information

Supplementary Figure 1. Linearity of the dilutions in terms of the concentration of Aβ aggregates. Since the brain homogenate corresponds to a suspension of a heterogenous mixture of Aβ aggregates, we calculated the concentration of total Aβ in various 2-folds dilutions performed in 3 different independent replicates. Aβ concentration was estimated by ELISA after incubation with formic acid to monomerize the protein. Data shows the average ± SEM and was fitted to a lineal regression with $r^2 = 0.9652$.

Supplementary Figure 2. Accumulation of Aβ-reactive amyloid aggregates in brain regions other than hippocampus or cortex. Representative 4G8 staining of animals in the groups injected with 10^{-1} and 10^{-3} dilution and sacrificed at 285 days old as well as in non-treated animals sacrificed at 19-21 months old. Pictures show representative images of thalamus and olfactory bulb. The bar in the top left panel corresponds to 100 μm and applies to all pictures.

Supplementary Figure 3. Lack of staining for hyperphosphorylated tau in experimental animals. Representative slides from the hippocampus and cortex of animals injected with the 10^{-1} dilution of brain extract as well as non-treated animals sacrificed at 9 or 21 months old, were stained with the AT8 antibody, which recognizes hyperphosphorylated tau protein. As positive control a slide from the cerebral cortex of a confirmed case of AD was stained at the same time (top right) and depict the characteristic staining of hyperphosphorylated tau in neurofibrillary tangles. The bar in the top left panel corresponds to 100 μm and applies to all pictures.

Supplementary Figure 1

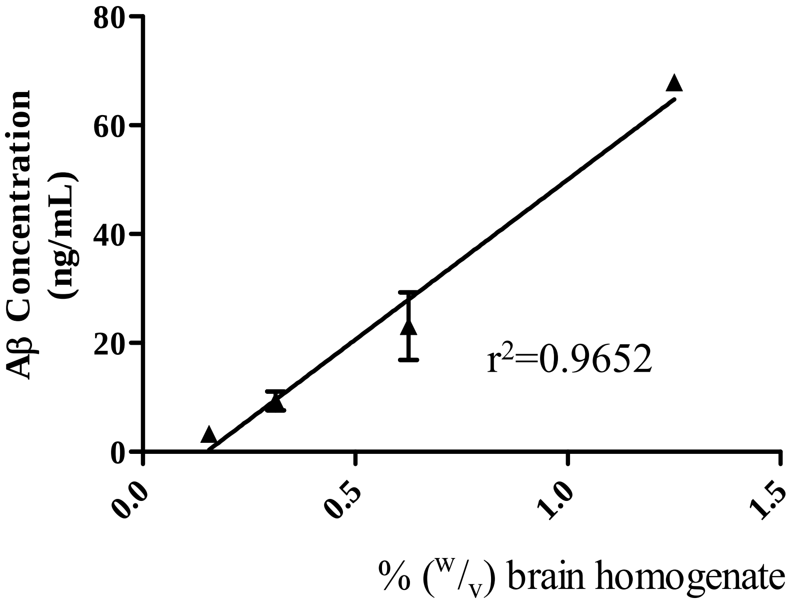

Supplementary Figure 2

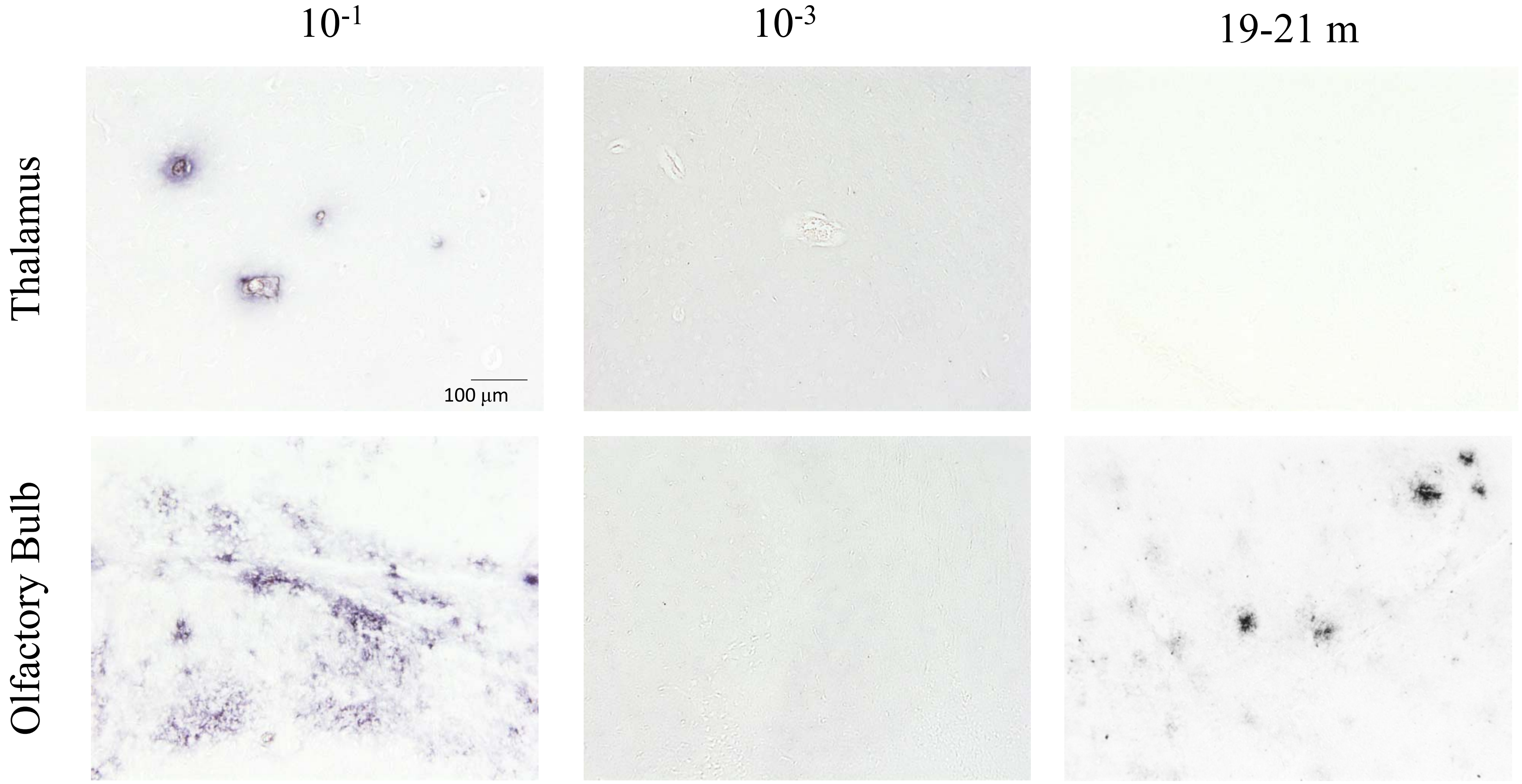

Supplementary Figure 3

Cortex AD Patient
AT8

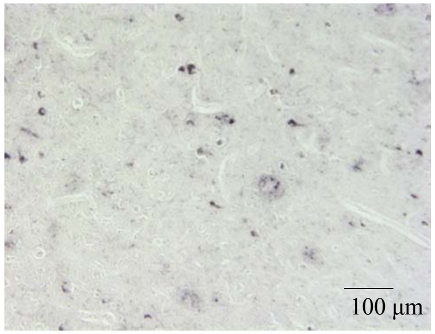

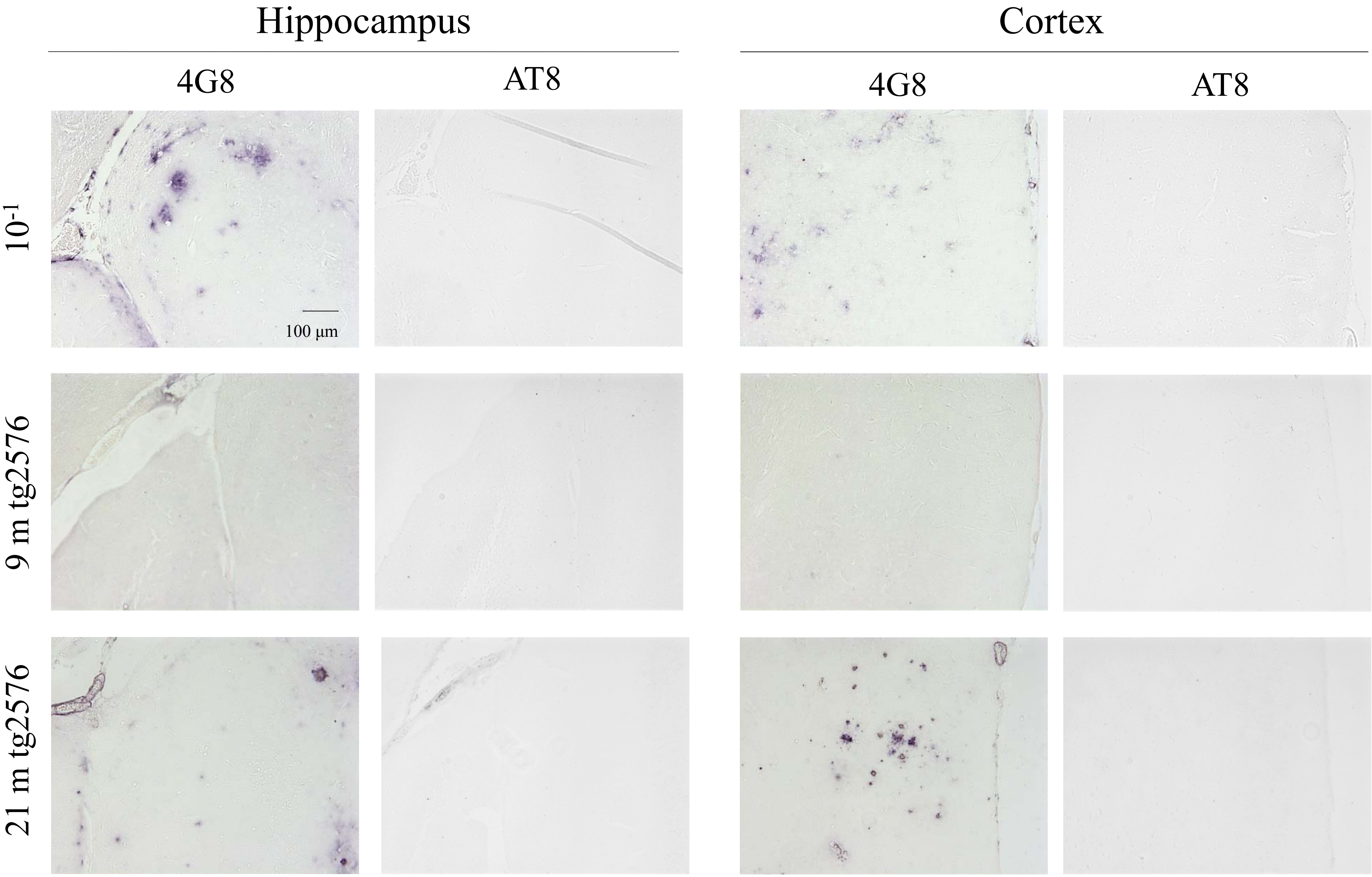

Supplement: Supplementary Information — Supplementary figures [file srep09349-s1.pdf]
